# A reappraisal of the phylogenetic placement of the *Aquilegia* whole-genome duplication

**DOI:** 10.1186/s13059-020-02212-y

**Published:** 2020-12-08

**Authors:** Tao Shi, Jinming Chen

**Affiliations:** 1 Key Laboratory of Aquatic Botany and Watershed Ecology, Wuhan Botanical Garden, Chinese Academy of Sciences, Wuhan, 430074 China; 2 Center of Conservation Biology, Core Botanical Gardens, Chinese Academy of Sciences, Wuhan, 430074 China

## Abstract

The accurate placement of an ancient whole-genome duplication (WGD) in relation to the lineage divergence is important. Here, we re-investigated the *Aquilegia coerulea* WGD and found it is more likely lineage-specific rather than shared by all eudicots.

Whole-genome duplications (WGDs) are frequent and common in plants, contributing to the evolutionary novelty and adaptation to extreme environments [1, 2]. Aköz and Nordborg reported a common WGD in the ancestral eudicot: while this ancestral tetraploid is preserved in *Aquilegia* (a basal eudicot), the hexaploid in the ancestral core eudicot was formed by hybridization of this tetraploid and another diploid with a subsequent WGD [3]. Nevertheless, this is contradictory to the studies of *Nelumbo nucifera* [4, 5]. *Nelumbo*, another basal eudicot, having a much closer relationship with core eudicots [6], shows a slower synteny loss and substitution rate than *Aquilegia* and core eudicots when aligned to outgroup species including *Nymphaea colorata* [7], rice, and *Brachypodium distachyon* (Figs. 1b and 2e) [4], and thus, *Nelumbo* should preserve more of its traces. However, the *Ks* peak corresponding to the “shared WGD” is absent in *Nelumbo*, and only a lineage-specific WGD after the *Nelumbo*-*Macadamia* split was found [4] (Fig. 2a), which raises doubt about their hypothesis of the common tetraploid origin.

**Fig. 1:**
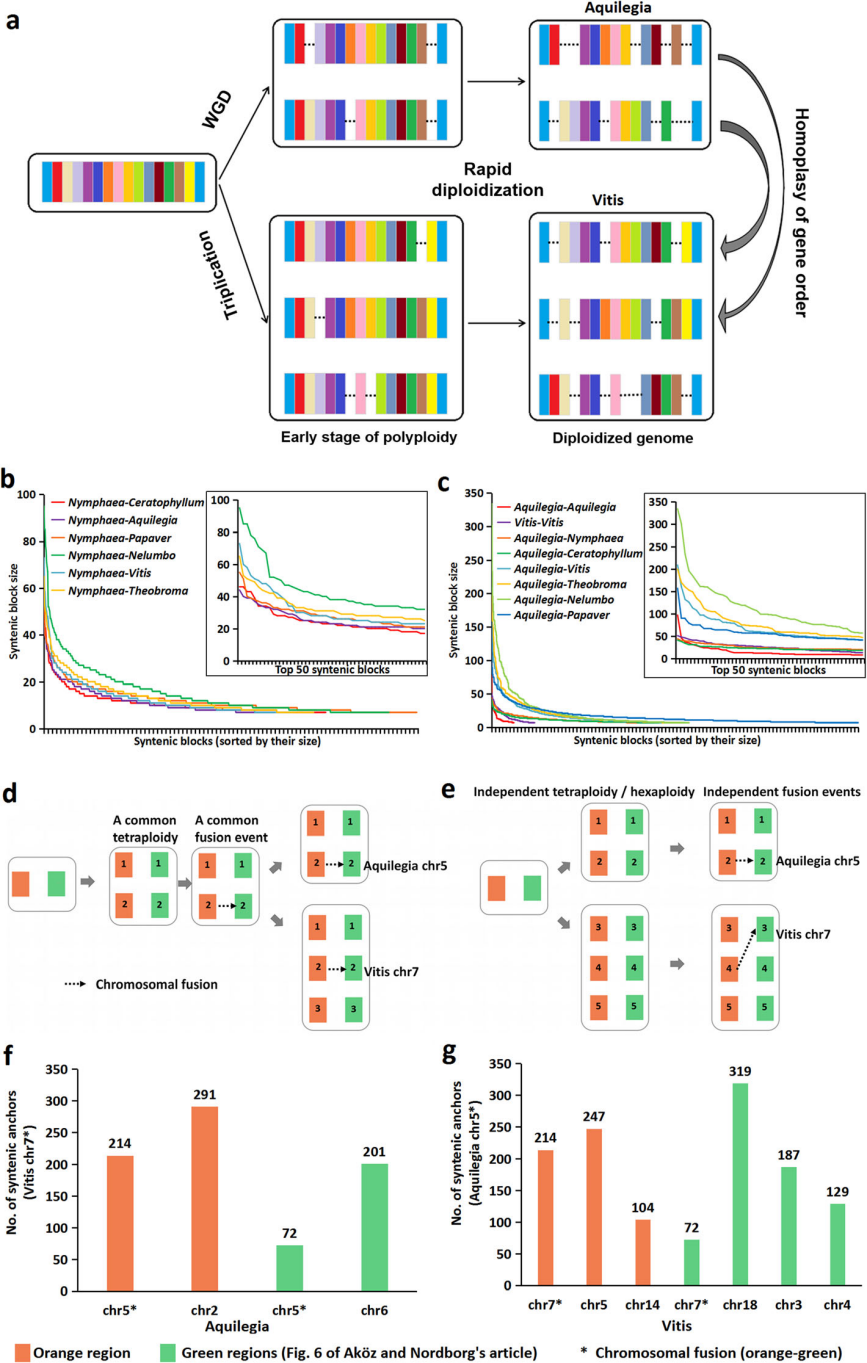
Genome structural analyses of *Aquilegia* and related species. **a** Scenario of rapid intraspecific synteny loss (diploidization) causing homoplasy of interspecific syntenies. **b**, **c** Distribution of interspecific and intraspecific syntenic blocks according to syntenic block size. **d** Scenario of a chromosomal fusion in the common ancestor of *Aquilegia* and *Vitis*. **e** Scenario of two independent chromosomal fusions in *Aquilegia* and *Vitis*, respectively. **f**, **g** Number of syntenic anchors between the orange-green fused chromosomes (*Vitis* chr7 and *Aquilegia* chr5) and their corresponding homologous regions

**Fig. 2:**
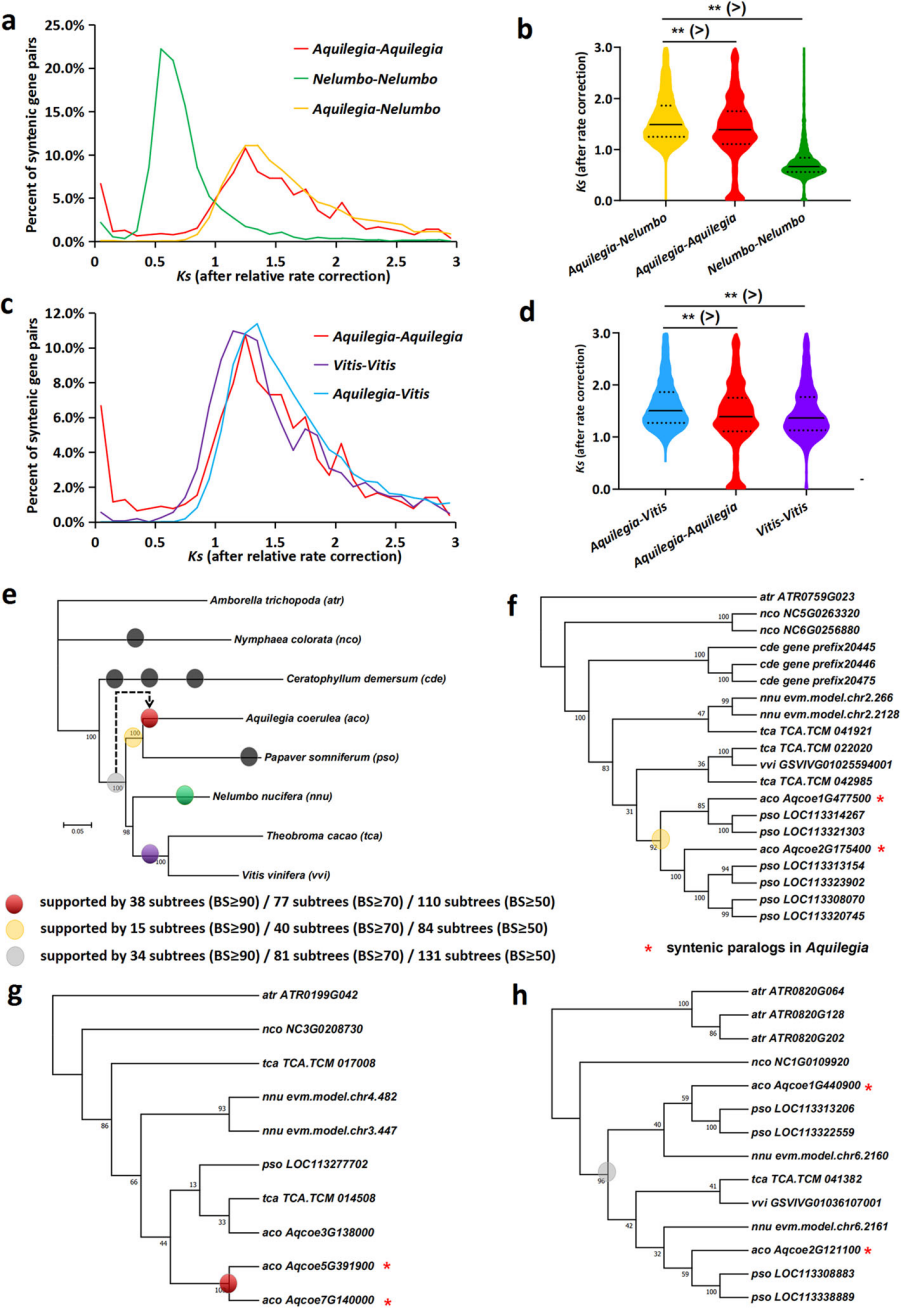
Phylogenomic analyses of syntenic duplicates from *Aquilegia*. **a**–**d** Histograms and violin plots of rate-corrected *Ks* of gene pairs from intraspecific and interspecific synteny in *Aquilegia* and *Nelumbo* (**a**, **b**) and in *Aquilegia* and *Vitis* (**c**, **d**). Median *Ks* values are marked by the black line in violin plots. **p value < 0.0001. **e** A species tree based on a concatenated alignment of 697 strict single-copy genes. **f** Example gene tree supports the hypothesis of *Aquilegia* WGD only shared by *Aquilegia* and *Papaver*. **g** Example gene tree supports the hypothesis of *Aquilegia* WGD being *Aquilegia*-specific. **h** Example gene tree supports the hypothesis of *Aquilegia* WGD occurring in the ancestral eudicot. Node values: bootstraps. Branch length: the number of amino acid substitutions per site. Colored dots: WGD or triplication. Arrow: the difference in placement of the *Aquilegia* WGD

The authors’ assertion that the *Aquilegia* WGD is shared by all eudicots is mainly based on clustering by gene order similarity or structural similarity within species genome and between species, which depends on synteny evolutionary rate. When the trait evolves with rate heterogeneity, the simple clustering by similarity can hardly reflect the true phylogeny. Therefore, the more rapid loss of intraspecific synteny in *Aquilegia* might cause homoplasy such that higher similarity in gene order of *Aquilegia-Vitis* than *Aquilegia-Aquilegia* can be observed (Fig. 1a). Indeed, *Aquilegia*-*Aquilegia* shows the most rapid decay of synteny with the smallest size and the fewest number of syntenic blocks, which is even smaller and fewer than older divergent pairs (*Aquilegia*-*Ceratophyllum* and *Aquilegia*-*Nymphaea*) (Fig. 1c) [7]. Rapid convergent loss of the alternative copies, particularly housekeeping genes, by selection against redundancy after WGDs was found in angiosperms [8], and this is also congruent with more rapid syntenic gene loss that occurred soon after a WGD in teleost fish [9]. Therefore, their placement of a WGD simply by gene order similarity is flawed because of homoplasy caused by the heterogeneous rates of synteny loss. Meanwhile, the authors claimed that a fusion of ancestral eudicot chromosomes “green” and “orange” was observed in both *Aquilegia* chr5 and *Vitis* chr7, which is derived from a fusion in their tetraploid ancestor (Fig. 1d). Yet, they did not exclude an alternative possibility of two independent green-orange fusions in *Aquilegia* and *Vitis*, respectively (Fig. 1e). If their assumption is true, the fused green-orange chromosomes are expected to share closer homology (more syntenic anchors) to each other than the other green or orange regions. On the contrary, we found that the green and orange regions in the fused *Vitis* chr7 and *Aquilegia* chr6 share more anchors with non-fused green or orange regions (Fig. 1f, g). Therefore, the scenario of two independent chromosomal fusions is more likely.

Here, we showed that the *Aquilegia* WGD is more likely lineage-specific rather than common to all eudicots by two different phylogenomic approaches. (1) *Ks* values of syntenic paralogs and orthologs were measured to infer the sequential order of WGD and species divergence. *Ks* of each lineage were corrected using the relative rate (*Aquilegia* to *Nelumbo* to *Vitis* = 1:0.750:0.970) based on *Ks* branch length ratios of 1425 single-copy ortholog groups [10]. For comparison, *Ks* > 3 were removed to prevent substitutional saturation [11]. The *Ks* distances of syntenic orthologs for both *Aquilegia-Nelumbo* and *Aquilegia-Vitis* are significantly longer than intraspecific paralogs in *Aquilegia* (Mann-Whitney *U* test, both *p* value < 0.0001), suggesting this WGD exclusively occurred in *Aquilegia* (Fig. 2a–d). (2) To circumscribe *Aquilegia* syntenic duplicates in relation to the species divergence, 697 gene trees of ortholog groups from key taxa containing the *Aquilegia* syntenic paralogs were reconciled with the species tree using Notung2.9 (www.cs.cmu.edu/~durand/Notung) [12–14]. The species tree was constructed from 176 strict single-copy genes by OrthoFinder (www.stevekellylab.com/software/orthofinder) and IQ-TREE (www.iqtree.org) (Fig. 2e). When we applied bootstrap thresholds of 90, 70, and 50, we found the hypothesis that *Aquilegia* syntenic duplications occurred after the divergence of *Aquilegia* and the ancestor of *Nelumbo* and core eudicots is supported by 53, 117, and 194 subtrees, respectively (red and yellow dots in Fig 2e–g), whereas hypothesis that duplication occurred in the ancestral eudicot is supported by 34, 81, and 131 subtrees, respectively (gray dot in Fig. 2e, h). Due to the fact that *Aquilegia* WGD and *Aquilegia*-*Papaver* split are closely spaced in time, it is more difficult to resolve their sequential order. However, more subtrees support *Aquilegia* WGD being independent (red dot in Fig. 2e, g) than being shared by *Aquilegia* and *Papaver* (yellow dot in Fig. 2e, f), which is in line with a 2:2 syntenic relationship between *Aquilegia* and *Papaver* [15].
